# Dendra2 Photoswitching through the Mammary Imaging Window

**DOI:** 10.3791/1278

**Published:** 2009-06-05

**Authors:** Bojana Gligorijevic, Dmitriy Kedrin, Jeffrey E Segall, John Condeelis, Jacco van Rheenen

**Affiliations:** Department of Anatomy and Structural Biology, Albert Einstein College of Medicine - Yeshiva University; Gruss Lipper Biophotonics Center, Albert Einstein College of Medicine - Yeshiva University; Hubrecht Institute-KNAW and University Medical Center Utrecht

## Abstract

In the last decade, intravital microscopy of breast tumors in mice and rats at single-cell resolution^1-4^  has resulted in important insights into mechanisms of metastatic behavior such as migration, invasion and intravasation of tumor cells^5, 6^, angiogenesis^3^ and immune cells response^7-9^. We have recently reported a technique to image orthotopic mammary carcinomas over multiple intravital imaging sessions in living mice^10^. For this, we have developed a Mammary Imaging Window (MIW) and optimized imaging parameters for Dendra2^11^ photoswitching and imaging *in vivo*. Here, we describe the protocol for the manufacturing of MIW, insertion of the MIW on top of a tumor and imaging of the Dendra2- labeled tumor cells using a custom built imaging box. This protocol can be used to image the metastatic behavior of tumor cells in distinct microenvironments in tumors and allows for long term imaging of blood vessels, tumor cells and host cells.

**Figure:**
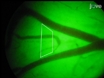


## Protocol

### 1. Generation of fluorescent tumors using injection of tumor cells into the mammary fat pad:

Grow a cell line expressing Dendra2 protein (as a cytoplasmic marker) to 40 - 80% confluency.
Rinse dishes at least 3 times with PBS w/o Ca^2+^ or Mg^2+^.
Add 3 ml of trypsin per 10 cm dish and incubate at 37 °C until most of the cells detach and then hit the dish against a flat surface to shake it.
Rinse all the cells off the dish, and use a scraper (rubber policeman) to collect matrix as well.  Add the 5 ml of PBS. Take an aliquot to count during centrifugation.
Centrifuge at 800 g for 5 min.
Aspirate and resuspend in PBS to a concentration of 5- 10x 10^6/^ml.  Store on ice until injected (inject within 30 min).
Place a cage containing 4-5 week old female immune deficient mice (e.g. SCID) inside a sterile hood.
Spray the area around the 4th (abdominal) nipple with 70% ethanol.
Inject 0.1 ml into mammary fat pad. If an assistant can be found, one person can hold the mouse in place while the other inserts the needle inside the mammary fat pad. If you are injecting by yourself, put the mouse under light anesthesia using isoflurane.
Once the tumor has grown to 5-7 mm, the Mammary Imaging Window (MIW) should be inserted.   Note: Alternatively, for some applications, the MIW can be inserted on top of the healthy mammary fat pad and the cells can be injected afterwards. That approach allows imaging of transiently transfected cells in a physiological environment.

### 2. Manual fabrication of the Mammary Imaging Window (MIW):

MIWs (Figure 1A) are made of tissue grade plastic to guarantee biocompatibility. For manual construction, we use 5 or 10 cm dishes.
Put latex gloves on.
Heat up a tissue-culture dish and creat a curved surface by pushing a rounded, hard object against the heated dish (we use a 1-inch dremmel bit for this).
Cut out the center of the dish using a heated razor blade.
Use a small, cone-shaped dremmel bit to make a hole in the center of the dome-shaped plastic base (6-7mm in diameter).
Make the edges of the plastic base completely smooth by sanding it with the dremmel and further filing it.
File the top of the plastic base making a flat surface for the glass coverslip. The diameter of the filed, flat  surface should be 9-10mm.
Glue the 8mm circular glass coverslip (number 1) on the flattened surface using the superglue (cyanoacrylate adhesive).
Wait for the glue to dry (15 minutes).
Use a heated 26G needle to make eight suturing holes puncturing from the outside (where the glass is) to the inside of the base. Holes should be evenly distributed around the coverslip, .5-1mm away from the edge of the coverslip.
Widen the holes using a 5-0 suturing needle.
Puncturing holes in the plastic base will make the inner surface uneven. Using sand paper, make this surface completely smooth.
Change the gloves and brush of small plastic particles from the MIW using a small brush.
Wash the MIW with deionized water.
Wash the MIW using 70% ethanol. Use a Q-tip to clean the glass so it is completely transparent. If there are foggy spots present from the glue vapor, carefully use acetone with a Q-tip.
Sterilize the MIW by UV exposure for 3 h at each side.

### Semi-Manual fabrication of the Mammary Imaging Window (MIW):

In order to fabricate the plastic base, we are currently using silicone rubber casting molds created using hand-made MIWs. The mold is comprised of two parts of silicone rubber that make an exact replica of both the front and back of the original when filled with polyester resin and then immediately joined together. The liquid polyester resin is mixed together 9:10 and results in a hard structure when fully cured in 48h. The plastic is UV protected and archival without breaking down or becoming yellow over time. After the base is cured, steps 2.8-2.16 are done the same way as for manually-made MIWs.

### 3. Insertion of the MIW:

The area around the 4^th^ nipple should have a small (5-7 mm diameter) tumor several days/weeks after injection of tumor cells depending on the cell type (Figure 1B). How much time has passed after injection of cells expressing Dendra2 photoswitchable protein or other fluorescent proteins depends on the cell line used. The tumor should not be visibly necrotic, and should have an intact skin with hair on top of the tumor. Mice with necrotic tumors should be euthanized.
Prepare the sterile space for the surgery- lay down a piece of sterile cloth inside a sterile hood. Put a piece of sterile gauze in the middle, and lay a few MIWs and sterile instruments around it:  we use small standard scissors, spring scissors, microdissecting tweezers, microdissecting forceps, needle holder. Keep sterile Q-tips, a bottle of 70% ethanol and sterile gloves in the corner of the hood.
The mouse is anesthetized in the sterile hood using IP injection of 2.5% avertin (20 μl/g) in HBSS or, alternatively 10 μg/kg ketamine + 10 μg/g xylazine (contact your Animal Care Institute for approvals and ordering). Note: Avertin solution should be prepared bi-weekly, filter sterilized using a 0.22 μm filter and stored as 500 μl aliquots at 4 °C (in the dark).
Remove hair by shaving the area above the tumor using a small animal shaver.
Remove the rest of the hair using Nair hair removal cream (available in any drugstore). Clean the skin using ethanol-dipped Q-tips.
Transfer the animal onto the sterile gauze and apply ophthalmic ointment to the eyes to keep them from drying and infection.
Sterilize the skin with betadine and clean with 70% ethanol.
Transfer the animal onto a sterile surgical cloth inside the sterile hood.
Pull the skin immediately medial to the nipple using forceps and cut ~2mm incision in the skin.
Separate the underlying mammary fat pad from the skin using dissection scissors and forceps. The skin and hole stretch during this separation step to a size that will accommodate insertion of the MIW.
If accidently a vessel is hit during sugery, use a sterile Q-tips to remove any blood that is produced/to stop the bleeding from becoming excessive.
Insert the MIW such that there is skin on top of the MIW base and suture in place using non-absorbable thread and reverse cutting needle.
Use tissue adhesive or cyanoacrylate to fill in the suturing holes and secure the MIW to the skin.
Add TMP-SMX antibiotic mix: (Sulfamethoxazole 0.6 mg/ml, Trimethoprim 0.12 mg/ml) into the cage water bottle for 3 days before and after the surgery.
The animal stays under anesthesia for 1-4h after avertin IP injection. Since the surgery commonly takes ~45min, it is important to help the animal recover afterwards:
Place a hand heating pad (37 °C) (available in drugstores) on the bottom of the cage and cover it with gauze.
Keep the animal on top of the pad until it turns on its stomach and starts walking.
If the animal is unconscious for more than 3h, inject 0.3 ml of HBSS intraperitoneally for rehydration.
The animal is allowed to recover over the next 3-4 days before the first imaging session takes place.

### 4. Imaging Box Construction

The imaging box assures that the MIW is sitting flat, right above the microscope objective. It also facilitates temperature and anesthesia control through constant airflow of isoflurane. The box is made of plexiglass and glued together using plastic weld. It is fitted for the specific microscope stage and therefore the shape of its bottom varies depending on the microscope used (Figure 2 shows the scheme of the box fitted to Leica SP5 microscope stage). Dimensions of the box in centimeters are l=11.4 cm, w=7.6 cm, h=4.4 cm (Figure 2A). The bottom of the box consists of a hollow plate, 12.7x8.4 cm in size, which fits inside the Leica SP5 microscope stage, and two sliding “doors”, 5.7 X 5.7 cm_each, which form a circular opening (d=2.22 cm) when closed. The MIW fits into this opening. The front piece contains the inlet hole for the anesthesia delivery while one of the side pieces contains an outlet hole which leads to the vacuum. Note that the condenser and the slide holder need to be removed prior to the imaging box placement.

### 5. Imaging Box Use

Place the animal under anesthesia with isoflurane and apply ophthalmic ointment to the eyes.
Attach the isoflurane exhaust from the anesthesia machine to the imaging box front, on the outside of the inlet hole (Figure 2B).
Attach a second tube to the outlet hole of the imaging box. This tube should be attached to the gas filter and then to the vacuum.
Open the box lid and open the box doors and place the animal, MIW side facing the bottom, and  sliding the doors into the box.
Hold the box and adjust the bottom doors so that the MIW base is held and immobilized between them. The MIW base should be at the same level as the imaging box doors, while the MIW coverslip should be just a few millimeters below this level. Make sure that the positioning of the MIW between the bottom sliding doors ensures that the coverslip (glass portion of the MIW) lies flat, parallel to the bottom doors the box. This will ensure proper focusing.
Lower the microscope objective, to allow space for placement of the box on top of the microscope stage.
Place the box on the microscope stage, and adjust the stage so that the MIW coverslip is above the objective.
Focus the objective and start imaging.
While using isoflurane, the animal should be monitored by visual inspection of the breathing frequency. This can be done visually, or by monitoring the frequency of breathing artifacts appearing during imaging collection (these can interfere with data acquisition). The MouseOx pulse oximetry system (Starr life Sciences Corp) has been used succesfully. That way the data can be gathered continuously without turning the lights on in the room to check on the animal. Note: After each imaging session, animal recovery should be facilitated by wrapping a small hand heating pad in gauze or kimwipes and putting it under the animal in the cage. Note: If an immersion objective is used to image through the MIW, the immersion medium (glycerol, water, oil) should be cleaned off the imaging window. The MIW should be inspected for any cracks or other damage that might have occurred during imaging. This is critical for collecting data during multiple imaging sessions.

### 6. Photoswitching and imaging of Dendra2-labeled cells

The procedure is described for the Leica SP5 confocal microscope; the laser power at the focal plane, available objectives and software options vary when using other confocal microscopes or multiphoton imaging set up, and therefore the protocol described below should be used as a guide to optimize the experiment on your microscope.

By observing the tumor through the eyepiece (10x) with the green fluorescence filter, locate major blood vessels which are visibly flowing and laying flat in the same focal plane.
Position one of the vessels in the center of the field and switch to PMT detection.
Set up sequential imaging collection for two channels. One of the channels uses 488nm laser line (10% power), and collects scattering from extracellular matrix (480-495nm) and emission from the green form of Dendra2 (505-540nm). The second channel uses the 543nm laser line (90% power) and collects emission from the red form of Dendra2 (555-600nm). Collect 3D images pre-photoswitching.
Use the ROI scan option to photoswitch a chosen population of cells using the 405nm laser line (30% power, 20-40 scans). Collect emission of the red form of the protein to monitor the switching.
Using the same sequential routine as in 6.3, collect post-switching 3D images.
If photoswitching more than one region in the tumor, it is advised to take pictures through the oculars using a digital camera, in both green and red channels (Figure 3A, B). This will help in the orientation during the subsequent imaging sessions. Note: Additionally, blood vessels can be temporarily labeled using tail-vein injection of fluorescent dextran (Cascade Blue or Alexa Fluor 647, both 10kDa). A few hours after the injection, dextrans will leave the blood vessels and permanently label the macrophages.

### Representative Results:

Figure 3C shows a rectangular photoswitched region (red) oriented orthogonally relative to the blood vessel (no fluorescence). Non-photoswitched cells are green, while the scattering from the extracellular matrix is purple. Image is a maximum intensity projection of four images along the Z-axis (20-50 μm depth).
